# Health Literacy and Emotional Management in Patients on Renal Replacement Therapy: A Mixed-Method Study Protocol

**DOI:** 10.3390/healthcare13091048

**Published:** 2025-05-02

**Authors:** Carmen González-Galán, Miriam Poza-Méndez, Martina Fernández-Gutiérrez, Pilar Bas-Sarmiento

**Affiliations:** 1 The Andalusian Health Service, Punta de Europa University Hospital, 11207 Algeciras, Spain; carmen.gonzalez.galan@hotmail.com; 2 Institute of Research and Innovation in Biomedical Sciences of the Province of Cadiz (INiBICA), 11009 Cadiz, Spain; pilar.bas@uca.es; 3 Department of Nursing and Physiotherapy, University of Cadiz, 11009 Cadiz, Spain; miriam.poza@uca.es; 4 The University Research Institute for Sustainable Social Development (INDESS), 11406 Jerez de la Frontera, Spain

**Keywords:** renal replacement therapy, health literacy, emotional management, adherence, quality of life

## Abstract

**Background:** Patients on renal replacement therapy (RRT) must adapt their lives to dialysis treatment, self-care routines, and medical restrictions, which can significantly impact their quality of life and emotional wellbeing. Additionally, limited health literacy hinders adherence to self-care practice, negatively affecting their health outcomes and treatment effectiveness. Given these challenges, this study aims to design and develop an action protocol that involves a change in the approach to health literacy and emotional management for patients on RRT, tailored to their specific needs, considering the different perspectives that influence the patient and their environment, including organizational, structural, care, and relational dimensions. **Methods:** The study will be based on the Ophelia (Optimizing Health Literacy and Access) methodology and consists of three phases over a three-year period (2025–2027): (a) a mixed design to explore health literacy level, coping strategies, adherence to self-care, and quality of life; (b) the development of the action protocol based on scientific evidence and the needs detected in this population; and (c) the validation of the protocol content by an expert panel made up of patients, professionals, and managers. **Conclusions:** The expected outcome is a clinically applicable protocol designed to improve health literacy and emotional management in patients undergoing RRT. This protocol will support healthcare administrators in structural and organizational planning, assist providers in delivering comprehensive care, enhance emotional support, and promote health literacy among patients. Ultimately, it aims to improve treatment adherence, health outcomes, and quality of life for this population.

## 1. Introduction

Chronic kidney disease (CKD) is a public health problem [1] with a significant impact on patients’ quality of life. Patients with CKD undergo significant changes in their lives from the start of treatment: dietary and fluid restrictions, symptoms that affect their quality of life, and the incorporation of complex self-care, among others. Renal replacement therapy (RRT) imposes significant limitations on patients’ social, professional, and family lives, forcing them to restructure their daily routines around the disease. This treatment affects quality of life from a physical, psychological, and social perspective [2], with variations depending on the RRT modality, and patients on RRT experience a lower quality of life than the general population [3,4].

Other factors that can affect the quality of life of patients on RRT are sociodemographic variables (female sex, advanced age, low educational level, or low socioeconomic level), psychological variables (presence of anxiety or depression), associated comorbidities (diabetes, cardiovascular disease), time since CKD diagnosis and duration of RRT, the presence of symptoms (fatigue, pain, pruritus...), and dependence on a caregiver [3,4,5].

From a psychological point of view, patients on RRT experience multiple stressors that influence their chosen coping style. These include RRT itself, pharmacological treatments and side effects, dietary restrictions, dependency, sexual dysfunction, loss of employment, comorbidity, time limitations, limitations in functionality, loss of roles, effects of the disease, and fear of death [6]. The identification of coping strategies in these patients is a topic that has been rarely explored in the literature. After an extensive review of the scientific literature, only two cross-sectional studies and one narrative review were found [7,8,9], indicating that the most commonly used coping strategies include information seeking, problem solving, cognitive restructuring, delegation, and the regulated expression of emotions. Furthermore, it was observed that approach-oriented strategies were used more frequently than avoidance-oriented strategies [8,9]. Moreover, the perception of the disease significantly influences the selection of coping strategies and can serve as a key predictor of a patient’s willingness to engage in self-care behaviors [7].

Similarly, patient adherence to treatment and self-care is determined by several key factors, including a realistic assessment of their knowledge and understanding, clear and effective communication between healthcare professionals and patients, and the fostering of a therapeutic relationship based on trust. Understanding the patient as an individual enables healthcare professionals to better identify and address the elements crucial for adherence [10].

Regarding self-care adherence rates among patients on RRT, most existing studies focus on hemodialysis patients, evaluating adherence to dietary and pharmacological therapy. However, the results are highly heterogeneous and vary significantly, often relying on subjective measures [11].

### 1.1. Health Literacy in Patients on Renal Replacement Therapy

Health literacy (HL) is particularly important in the context of CKD due to the complexity of the disease, its comorbidities, and its treatment [12]. According to Sørensen et al. [13], HL is defined as “the knowledge, motivation, and skills of individuals to access, understand, evaluate, and apply health information in order to make decisions in everyday life related to the promotion and maintenance of health, disease prevention, and health care”. Initially linked to basic individual competencies in the context of medical care, this concept has evolved significantly to incorporate organizational structures and sociocultural contexts [14].

In CKD, the prevalence of inadequate HL ranges from 5% to 60% [15,16,17]. Low HL in CKD patients has been associated with a reduced estimated glomerular filtration rate at baseline [15] and unfavorable cardiovascular risk profiles [18]. Patients with low HL levels exhibit poorer adherence to dialysis, an increased incidence of cardiovascular events, higher mortality rates, worse clinical outcomes, and greater utilization of healthcare resources [12,16,17,19]. Additionally, low HL is linked to limited knowledge of one’s health status, reduced participation in self-care, and a greater disease burden [16].

Several studies have demonstrated the relationship between adequate HL levels and improved self-care behaviors, quality of life, and clinical outcomes [12,20]. However, the assessment of HL has primarily focused on functional competencies, such as health-related literacy and numeracy [21], often neglecting more complex dimensions, including critical and communicative skills, which are crucial for CKD self-management [22]. Therefore, it is necessary to develop targeted interventions that align with the health literacy level and the needs of patients, and that assess their impact on variables such as adherence and quality of life [23].

### 1.2. Emotional Management in Patients on Renal Replacement Therapy

The evaluation of how RRT affects emotional wellbeing is based on studies focusing on the detection, prevalence, and treatment of depression and anxiety. The evaluation of the presence of these two psychological conditions is indicated as a recommendation for healthcare providers [2].

Although the evidence remains limited, various interventions have been identified, including psychoeducation, relaxation techniques, mindfulness, and the integration of a psychologist into the professional team, all of which may improve emotional wellbeing, treatment adherence, and quality of life in these patients [2,24].

Despite advancements in healthcare for patients undergoing RRT, significant gaps persist in the comprehensive understanding of their lived experiences and in the development of integrated approaches that address both health literacy (HL) and emotional management. These two dimensions are critical to improving health outcomes in this population. To date, no protocol has been identified that concurrently addresses both HL and emotional management in patients receiving RRT.

The purpose of this project is to design and develop an action protocol that involves a change in the approach to health literacy and emotional management for patients on RRT, tailored to their specific needs, considering the different perspectives that influence the patient and their environment, including organizational, structural, care, and relational dimensions.

The specific objectives proposed are as follows:

- To identify the level of health literacy and coping strategies of patients on RRT, explore quality of life, and assess adherence to self-care, considering gender differences.
- To define good practices based on scientific evidence and the needs detected in patients on RRT, focusing on the approach to health literacy and emotional management.
- To design and validate the content of a protocol, framed within a procedure that involves the organization, healthcare professionals, and patients, while incorporating a gender perspective and addressing the needs identified in this population.

## 2. Materials and Methods

**Figure 1:**
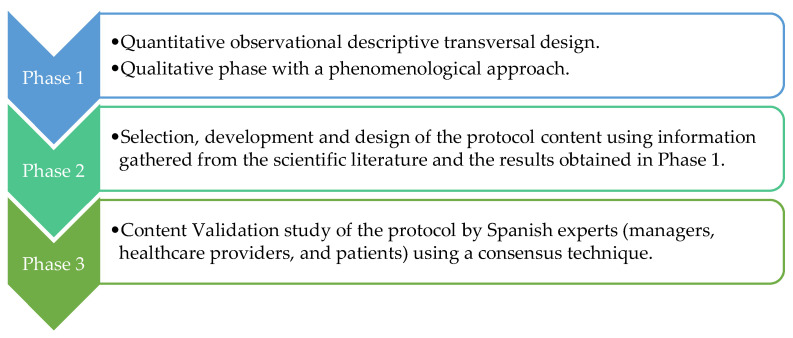
Phases study.

This will be a mixed study, divided into three phases (Figure 1), based on the Ophelia (Optimizing Health Literacy and Access) methodology [25]. The project timeline will cover the period from 2025 to 2027 (the timeline is detailed in Supplementary Materials Table S1).

### 2.1. Phase 1

#### 2.1.1. Study Design

A mixed design will be employed, comprising a quantitative, observational, descriptive, cross-sectional phase and a qualitative phase with a phenomenological focus. The needs of HL and emotional management in the population on RRT will be identified. Similarly, quality of life, coping strategies, and adherence to self-care will also be explored using quantitative strategies like self-informed questionnaires. In parallel, semi-structured individual interviews will be conducted with selected patients during their treatment sessions or coinciding with their follow-up visits to the health center. The goal in the qualitative phase will be to explore patients’ experiences and the causes or motivations that lead them to engage in certain behaviors, as well as to understand the reasons for non-adherence to self-care and the expectations or needs identified in the current approach to their HL and emotional management.

This phase 1 will be in accordance with the Guidelines for Conducting and Reporting Mixed Research in the Field of Counseling and Beyond [26], as recommended by the EQUATOR Network.

#### 2.1.2. Participants and Setting

The study will be conducted in healthcare centers in the province of Cádiz, Spain, that offer the selected RRT modalities: hemodialysis, peritoneal dialysis, and home hemodialysis. In addition, two peripheral hemodialysis centers will be included to ensure the representation of patients who do not receive treatment exclusively in the hospital setting.

The study population will consist of all patients on RRT in the province of Cádiz (a total of 382 patients), distributed according to the type of RRT: 304 on hemodialysis, 68 on peritoneal dialysis, and 10 on home hemodialysis.

#### 2.1.3. Inclusion and Exclusion Criteria

The study will include patients over the age of 18 who are enrolled in an RRT program at the time of data collection, who are able to engage in a conversation in Spanish, and who can read and understand documents in Spanish.

Patients with impaired verbal communication or diagnosed cognitive impairments will be excluded.

#### 2.1.4. Sample Size

For the cross-sectional descriptive observational quantitative analysis, proportional stratified sampling will be performed, based on the different RRTs and centers, as well as their representation in the study population. The sample size was estimated with a 95% confidence level and a 5% margin of error: 170 hemodialysis patients, 58 peritoneal dialysis patients, and 10 home hemodialysis patients.

For the qualitative design analysis, in which semi-structured interviews will be conducted, a purposive sampling strategy will be used to ensure the adequate representation of all the RTT modalities, with the initial selection of key informants. Additionally, the sample will be designed to account for diversity in terms of sex, educational level, and socioeconomic status. Qualitative data collection will proceed with as many participants as required until theoretical saturation is achieved, defined as the point at which data redundancy occurs and no additional relevant codes, themes, or dimensions emerge during analysis.

#### 2.1.5. Outcome Measures and Instruments

- Sociodemographic variables: Sex, age, country of origin, marital status, children, family unit, employment status, and educational level.
- Clinical variables: Diabetes, hypertension, comorbidities (heart failure, cancer, thyroid disorders), family history of kidney disease, modality of RRT, type of vascular or peritoneal access, number of sessions per week, time since initiation of RRT, previous transplant, or inclusion on the transplant waiting list.
- Health literacy level: The Spanish version of the European Health Literacy Survey Questionnaire (HLS-EU-Q16) [27] contains 16 items addressing self-reported difficulties in accessing, understanding, appraising, or applying the information to tasks related to making decisions in healthcare, disease prevention, or health promotion. Each item is rated on a four-point Likert scale (very difficult, difficult, easy, and very easy) and a “do not know/no answer”. “Do not know or no answer” answers are coded as “no answer”. Following the authors’ instructions, when scoring the HLS-EU-Q16, the categories “very difficult” and “difficult” are scored as 0, and the categories “easy” and “very easy” are scored as 1. Scale values are calculated as simply summed scores only for respondents who answered at least 14 items. Scoring varies between 0 and 16, establishing three levels of HL: inadequate (0–8), problematic (9–12), or sufficient (13–16).
- Variables related to quality of life: The Spanish version of the Kidney Disease Quality of Life-36 (KDQOL-SF™) [28] is a specific questionnaire for patients with kidney disease on dialysis. It consists of a general section (12 items) that evaluates physical function, physical role, emotional role, pain, mental health, vitality, social function, and general health, grouped into the global dimensions of physical and mental health. The specific part (items 13–36) deals with the burden of the disease, symptoms, and effects on daily life. Scores range from 0 to 100 per dimension and 0 to 400 for the overall dimensions, where higher scores indicate a better quality of life. The Spanish version was used.
- Variables related to coping/emotional management: The Spanish adaptation of the Coping Strategies Inventory Short Form (CSI-SF) will be employed [29]. The CSI-SF maintains the structure of the original instrument and comprises four subscales, each consisting of four items: (a) Problem-Focused Engagement (items 1, 2, 8, and 9), (b) Problem-Focused Disengagement (items 4, 7, 12, and 14), (c) Emotion-Focused Engagement (items 5, 6, 11, and 13), and (d) Emotion-Focused Disengagement (items 3, 10, 15, and 16). Participants will be instructed to indicate the general frequency with which they employ each coping strategy, using a Likert-type scale ranging from 1 (“Never”) to 5 (“Almost Always”), with intermediate values of 2 (“Seldom”), 3 (“Sometimes”), and 4 (“Often”).
  Scores will be computed for the two primary (first-order) dimensions—Engagement and Disengagement (score range: 8–40)—as well as for the four secondary (second-order) subscales—Problem-Focused Engagement, Problem-Focused Disengagement, Emotion-Focused Engagement, and Emotion-Focused Disengagement (score range: 4–20). The CSI-SF adopts a two-dimensional model of coping, categorizing strategies along two axes: cognitive-behavioral orientation (commitment vs. avoidance) and coping focus (problem-focused vs. emotion-focused). Internal consistency analyses have demonstrated high reliability across all subscales, and the psychometric evaluation of the Spanish version yielded satisfactory fit indices, supporting its validity as a robust measure of coping strategies in response to stress [29].
- Variables related to adherence to the self-care system: The Spanish version of the End-Stage Renal Disease Adherence Questionnaire (ESRD-AQ) [30] consists of 46 items divided into 5 sections: General Information (items 1–5), Attendance at Dialysis Treatment (items 6–19), Adherence to Drug Treatment (items 20–28), Adherence to Fluid Restriction (items 29–38), and Adherence to Diet (items 39–46). The answers to the questionnaire are made up of a combination of Likert-type scales, multiple-choice options, and yes/no answer formats. The measurement of overall adherence is scored by adding the scores of items 14, 17, 18, 26, 31, and 46. The scores are adjusted according to the reasons for non-adherence. For example, in item 14, “How many dialysis treatments did you miss last month?” and in item 15, “What was the main reason you did not attend your dialysis treatment?” The answer to item 14 may indicate a lack of adherence to treatment, but if the answer to item 15 is related to medical causes, then the score for item 14 is not reduced, as non-adherence is considered justified. In ESRD-AQ, higher scores indicate better adherence. Adherence to dialysis treatment is scored by adding the scores of items 14, 17, and 18. The maximum possible score is 600 (best adherence) and the minimum score is 0 (worst adherence). Adherence to pharmacological treatment corresponds to the score of item 26, with a maximum score of 200 and a minimum score of 0. Adherence to water restriction is scored with the score of item 31, with a maximum score of 200 and a minimum score of 0. Adherence to the diet is evaluated with the score of item 46, with a maximum score of 200 and a minimum score of 0. Overall adherence is the sum of all these scores, with a maximum of 1200, which would indicate the best possible adherence. In the questionnaire, as well as measuring adherence to self-care, information can be obtained about patients’ perception, attitude, and knowledge about the treatment and their self-care, as well as the frequency with which they receive information from healthcare personnel.
- Variables related to patients’ experiences. The design of the semi-structured interview will be informed by the integration of two complementary theoretical models: Ajzen’s Theory of Planned Behavior (TPB) [31] and Leventhal’s Common-Sense Model of Self-Regulation (CSM) [32]. This combined approach will be adopted to capture both the cognitive–rational dimensions of behavioral intention (TPB) and the emotional and perceptual representations of chronic illness (CSM), which are particularly relevant in the context of CKD. The Ajzen’s Theory of Planned Behavior [31] has been selected because it allows for the prediction and explanation of human behavior in specific contexts. According to Ajzen [31], behavior is influenced by three key factors: the individual’s attitude toward the behavior, perceived control, and social influence. Together, these factors determine the individual’s intention to carry out an action. In the context of this study, the Theory of Planned Behavior will offer a valuable framework for understanding patients’ behaviors in relation to dialysis treatment and self-care compliance. Applying this theory in this specific field will allow for an analysis of how these factors affect patients’ decisions and actions, contributing to a deeper understanding of their behavior and the identification of possible strategies to improve it. The CSM, developed by Leventhal and colleagues [32], is a theoretical framework that explains how individuals perceive, interpret, and respond to health threats. According to the CSM, individuals form cognitive and emotional representations of their illness, which guide their coping strategies and health-related behaviors. These representations typically encompass five key dimensions: identity (the label and symptoms of the illness), cause (perceived reasons for the illness), timeline (expected duration), consequences (anticipated effects), and controllability or curability (beliefs about the extent to which the condition can be managed or cured). In addition to these cognitive components, the CSM incorporates emotional responses, such as fear, anxiety, or sadness, which also influence coping behaviors. The model emphasizes a dynamic and iterative process, wherein individuals continuously evaluate the effectiveness of their coping strategies and adjust them accordingly. The CSM is widely used in chronic illness research, as it captures the subjective, experiential, and emotional aspects of living with a long-term condition [33]. To operationalize this theoretical integration, the interview guide will be organized into thematic blocks, each combining constructs from both models. This structure will allow for an in-depth exploration of how patients perceive their condition and make decisions regarding treatment and self-management.

#### 2.1.6. Data Analysis

This study follows a mixed-method design in which quantitative and qualitative data will be analyzed independently and subsequently integrated through a triangulation strategy.

Qualitative data will be analyzed using the program ATLAS.ti 8, following a narrative deductive content analysis to differentiate in the patients’ discourse the elements derived from the theoretical frameworks (the Theory of Planned Behavior and the Common-Sense Model of Self-Regulation). In parallel, an inductive narrative content analysis will be carried out to extract elements from the discourse that provide us with new information or ideas, which may support the detection of needs in the approach to health literacy and emotional management.

A descriptive analysis will be performed for all the quantitative variables, and an exploratory multivariate analysis will be conducted between the sociodemographic and clinical variables (independent) and the dependent variables resulting from health literacy, quality of life, coping, and adherence to self-care. SPSS Statistics version 23.0 for Windows (IBM) will be used. The statistical significance will be set at 95% (*p* < 0.05).

Prior to the analysis, the normality of the variables will be evaluated using the Kolmogorov–Smirnov test. Quantitative variables will be expressed as summary measures (means, medians) and dispersion measures (standard deviation, range), while categorical variables will be expressed in frequency and percentage. In the intergroup analysis, after the normality tests, for two independent samples, the Mann–Whitney U test will be used for non-parametric results, and the Student–Fisher *t*-test will be applied if the distributions of the variables are parametric. For the intragroup analysis of two related variables, the Student–Fisher *t*-test for parametric data or the Wilcoxon signed-rank test for non-parametric data will be used. To complement statistical hypothesis tests, Spearman’s correlation will be employed to analyze the strength of related linearity between paired data when the distribution between the variables is not normal, and Pearson’s correlation when the distribution is normal. Kruskal–Wallis or ANOVA tests will be used to test the hypothesis between variables with 3 or more categories and main variables.

In the final stage of analysis, triangulation will be employed to compare and contrast findings across both data strands. This process aims to enhance the validity, depth, and contextualization of the results by identifying areas of convergence, divergence, and complementarity. Through this integrative approach, we seek to generate a comprehensive understanding of the phenomenon under study, drawing on the strengths of both methodological paradigms.

### 2.2. Phase 2

The selection, development, and design of the contents of the action protocol for addressing HL and emotional management in patients on RRT is as follows.

The action protocol will be developed using the information obtained from the scientific literature and the results from phase 1.

#### 2.2.1. Study Design

A scoping review will initially be conducted with the aim of identifying evidence on activities, programs, projects, or interventions aimed at addressing HL or emotional management in populations with CKD.

This second phase of the protocol was drafted following the Preferred Reporting Items for Scoping Reviews Protocols (PRISMA-ScR) [34].

#### 2.2.2. Search Strategy and Study Selection

The following databases will be consulted for the scoping review: Cochrane Library, NICE, Web of Science, CINAHL, SCOPUS, PubMed, and Epistemonikos. The selection criteria will focus on meta-analyses, systematic reviews, experimental clinical trials, mixed designs with experimental trials related to best practices/interventions in HL, or emotional management for chronic kidney disease patients, with a focus on studies that report health outcome measures.

The search strategy will be guided by the MeSH Thesaurus, using controlled terms such as: “Health literacy”; “Coping”; “Adaptation, Psychological”; “Coping Behaviors”; “Coping Strategies”; “Renal Replacement Therapy”; “Chronic Kidney Disease”; and “End-Stage Kidney Disease”. Boolean operators such as “AND” and “OR” will be used to connect these terms.

From the studies identified, those meeting the inclusion and exclusion criteria will be selected (Table 1 and Table 2).

The concordance between the results of the scoping review and the needs identified in phase 1 will be analyzed to ensure alignment and to inform the design of the action protocol.

#### 2.2.3. Procedure

Following the Ophelia methodology [25], the activities to be included in the protocol will be selected based on the needs identified in the study population. This will include the definition of the needs detected, the expected objectives of the results, the proposed activities and the scientific evidence. The action protocol will address HL and emotional management from different perspectives surrounding the patient and their environment: organizational, structural, care-related, and relational.

Once the activities within the framework of the strategy for addressing HL and emotional management have been selected, the action procedure will be defined following the Ophelia methodology [25]:

Definition of the scope of application, entry limits, reach, and target audience of the procedure is as follows. 

Action planning: who, how, when, and with what resources/materials.

Prioritization of actions according to the degree of need, the urgency of action, the necessary resources, barriers, and facilitators.

Identification of roles and responsibilities of the implementation team.

Forecast timetable for the process of implementing the actions, and confirmation of resources.

Plan for evaluating the interventions using defined quality indicators.

Plan for disseminating the strategy in the centers involved and to the actors involved (patients—or caregivers in the case of older adults—professionals, managers).

Plan for dissemination to the public (patient associations, caregivers, scientific societies, universities, etc.).

### 2.3. Phase 3

#### 2.3.1. Study Design

This study will employ a content validation approach to assess the action protocol by experts using the consensus technique. A coalition of experts and key stakeholders involved in RRT will be assembled to ensure the comprehensive input and validation of the protocol.

#### 2.3.2. Participants and Setting

Sampling for the consensus technique will be carried out using non-probabilistic purposive sampling. A coalition of Spanish experts and key stakeholders in the field of RRT will be formed, comprising patients on RRT, nephrology care professionals, and managers of nephrology clinical units. To be eligible as an expert panel member, individuals must meet at least two predefined criteria corresponding to their respective category; failure to do so will result in exclusion from the study.

Patients undergoing Renal Replacement Therapy (RRT) must meet the following criteria: (a) receiving RRT for a minimum of one year; (b) active engagement in initiatives proposed by their nephrology unit; and (c) participation in peer-support programs. Healthcare professionals must fulfill at least two of the following: (a) a minimum of two-years’ experience in nephrology care; (b) participation in at least one implementation plan for quality improvement or patient care enhancement in nephrology; (c) involvement in research and/or scientific output in areas such as health literacy, emotional management, quality of life, adherence to self-care, and coping strategies in renal patients and/or validation of assessment instruments; or (d) a minimum of two-years’ teaching experience in the relevant field. For healthcare managers, they must meet at least two of the following: (a) a minimum of two years of experience in nephrology care and unit management; (b) participation in the design and implementation of quality plans, educational/research initiatives, or management agreements; (c) scientific production or research participation in the same thematic areas as healthcare professionals; or (d) at least two years of teaching experience in the relevant area.

In line with guidelines from the American National Institutes of Health and informed by previous consensus conferences in clinical settings [35,36], which typically involved expert panels of between 10 and 15 members, we estimate that the expert panel for this study will consist of 15 participants.

#### 2.3.3. Procedure

The experts will be tasked with evaluating the proposed protocol procedure, which will be designed based on the existing scientific evidence and structured around the following core components:

- Intervention;
- Activities;
- Responsibilities;
- Resources (digital approach);
- Barriers/facilitators;
- Evaluation indicators.

The procedure will undergo validation through a consensus conference, allowing experts to openly discuss and refine the proposed interventions and activities. Based on the discussions and contributions of the participants, a consensus will be sought on the final design of the procedure.

To this end, the preliminary document of the procedure will be presented to the experts, detailing the activities and strategies contemplated. During the conference, experts will have the opportunity to share their insights, opinions, needs, and concerns regarding the procedure. These contributions will be discussed and processed by the coalition to adjust and enhance the content. Particular attention will be paid to the strength of the evidence supporting each recommendation, and barriers and facilitators that could influence successful implementation will be thoroughly examined.

The entire conference will be systematically documented in the form of official minutes, which will also include the sociodemographic data of the participants. These data will be collected through a pre-conference questionnaire to contextualize and enhance the interpretation of their contributions.

At the conclusion of the consensus conference, a final document will be produced containing evidence-based recommendations for good clinical practice in addressing HL and emotional management in patients on RRT. These recommendations will be organized into specific activities and interventions designed to facilitate the implementation of the procedure and its subsequent evaluation. The resulting document will serve as a regulatory framework for the application of the procedure in the province of Cádiz, with the ultimate goal of improving patient care in RRT.

## 3. Discussion

HL and emotional management are pivotal components in the comprehensive care of patients on RRT. However, these dimensions remain insufficiently addressed in clinical practice and are not currently integrated into the management protocols for CKD within the Spanish Healthcare System. This study proposes an innovative strategy that combines both areas, tailored to the needs of the local population and the specific context of health services.

The Ophelia methodology, which forms the foundation of the protocol design, ensures that the intervention is aligned with the unique needs and characteristics of the target population. This approach enables the creation of a sequential protocol with personalized resources, roles, and activities, all underpinned by scientific evidence and validated by expert consensus.

The purpose of this project is to design and develop an action protocol that involves a change in the approach to HL and emotional management for patients on RRT, tailored to their specific needs, considering the different perspectives that influence the patient and their environment, including organizational, structural, care, and relational dimensions. The implementation of this project would allow us to address the existing gaps in the care of patients on RRT by providing the following: (a) guidance and support for healthcare professionals, including a compendium of best practices in addressing health literacy and emotional management; (b) strategies for nephrology service management to facilitate structural and organizational integration; and (c) tools and support to enhance the HL and emotional wellbeing of patients on RRT.

Integrating HL and emotional management into standard care protocols has the potential to transform nephrology services. Clinically, it is anticipated that this approach will lead to improved adherence indicators and enhanced quality of life for patients. Organizationally, this model could serve as a reference for implementing similar interventions across other units that care for chronic patients.

The limitations of this study include that this study concerns the context-specific nature of the protocol development, which will be tailored to the healthcare setting of the province of Cadiz (Spain). While this contextualization enhances the protocol’s local relevance and applicability, it may also limit its direct transferability to other regions or healthcare systems with differing organizational structures, resources, and patient populations. Additionally, the exclusion of patients with a diagnosed cognitive impairment—a practical decision to ensure the reliability of self-reported data collected through questionnaires and interviews—represents another limitation that could affect the overall scope and applicability of the proposed intervention framework. Finally, there are limitations related to the self-reported instruments used. The HLS-EU-Q16 collects the perceived difficulty in carrying out specific tasks and, therefore, does not objectively measure whether or not the person has the given competence. In this sense, the low HL may be due to a lack or deficiency in the population or a false perception by the individual of his or her competencies and skills or a health system with overly complex demands. Similarly, the results of the ESRD-AQ may be influenced by participants’ social desirability bias.

This study could have the potential to contribute to the theoretical framework by positioning HL and emotional management as interconnected pillars in the management of CKD. Furthermore, it establishes a foundation for future research to evaluate the effectiveness of implementing these best practices for this patient population.

## 4. Conclusions

This study could introduce an innovative approach to addressing the needs of patients on renal replacement therapy through the development of an action protocol that integrates both HL and emotional management.

The anticipated outcomes of this research will not only directly benefit the patients involved but will also provide a structured framework for transforming the approach to health literacy and emotional management within clinical settings. This represents a significant advancement toward a more patient-centered model of care, one that comprehensively considers the diverse needs of individuals in the context of CKD management.

## 5. Ethics Approval and Consent to Participate

This study adheres to the principles outlined in the Declaration of Helsinki, ensuring compliance with ethical standards that safeguard the rights, health, and dignity of all participants. The wellbeing of participants is paramount and takes precedence over all research objectives.

To uphold these ethical principles, data will be collected in a pseudonymized manner, in accordance with the seventeenth additional provision of Organic Law 3/2018 on the protection of personal data. A coding system will be used to pair data, and this system will be known only to the principal investigator, who is responsible for ensuring the confidentiality of the recorded data. Sociodemographic data will be self-reported by the patients.

All participants will be provided with an information sheet outlining the study and will be given a copy for their reference. Additionally, participants will be required to sign an informed consent form prior to participation. Participation in the study is entirely voluntary, and participants may withdraw at any time without any consequence.

Data will be stored on the University of Cadiz’s private cloud storage service (UCADrive), which includes encryption both in transit and at rest, user authentication, and access control. This service ensures secure, efficient, and reliable data storage, safeguarding data autonomy, security, and control. Upon the completion of the data retention period (a minimum of five years after the end of the study), the data will be permanently deleted using secure erasure techniques and the physical destruction of storage media.

The study has received approval from the Research Ethics Committee of the Province of Cadiz (code: SICEIA-2024-003056), ensuring that it meets the necessary ethical requirements for implementation.

## Figures and Tables

**Table 1:** Inclusion criteria.

Criterion	Description
Participants	Patients with chronic kidney disease on renal replacement therapy/patients with chronic kidney disease.
Study design	Meta-analyses, systematic reviews, experimental clinical trials, mixed designs with experimental trials.
Intervention	Interventions focused on health literacy or emotional management.
Setting	Hospital centers, healthcare facilities, or patient homes.
Outcomes	Measured outcomes in any of the following areas: health literacy level of the patient; emotional management or coping strategies; self-management/health management; quality of life. General health status: morbidity, quality of life, physiological indices, etc.
Publications	Scientific publications in databases such as Cochrane Library, NICE, Web of Science, CINAHL, SCOPUS, PubMed, and Epistemonikos.

**Table 2:** Exclusion criteria.

Criterion	Description
Participants	Patients with Acute Kidney Failure.
Study design	Quasi-experimental studies, observational studies (case-control, cohort, cross-sectional).
Intervention	Interventions not specifically designed to improve health literacy or emotional management. Interventions aimed solely at improving functional literacy (reading, writing, numeracy) for general populations.
Setting	Settings outside the healthcare or home environment of the patient.
Outcomes	Studies where the results are not related to those mentioned in Table 1 of the inclusion criteria.
Publications	Gray literature articles, press, or articles that cannot be acquired.

## Data Availability

Not applicable.

## Supplementary Materials

The following supporting information can be downloaded at: https://www.mdpi.com/article/10.3390/healthcare13091048/s1, Table S1: Project Schedule.

## Abbreviations

The following abbreviations are used in this manuscript:

CKD	Chronic kidney disease
ESRD-AQ	End-Stage Renal Disease Adherence Questionnaire
HL	Health literacy
HLS-EU-Q16	European Health Literacy Survey Questionnaire
KDQOL-36	Kidney Disease Quality of Life-36
Ophelia	Optimizing Health Literacy and Access
PRISMA-ScR	Preferred Reporting Items for Scoping Reviews Protocols
RRT	Renal replacement therapy
