# Supplementary material for: Health Literacy and Emotional Management in Patients on Renal Replacement Therapy: A Mixed-Method Study Protocol
Source: Healthcare (Basel). 2025 May 2;13(9):1048. doi: 10.3390/healthcare13091048 (PMC12071680; doi:10.3390/healthcare13091048)
Supplement: Supplementary file 1 [file healthcare-13-01048-s001.zip › healthcare-3566110-supplementary.pdf]

Table S1. Project Schedule

Task	Timeline																																		
	**1**	**2**	**3**	4	5	6	7	8	9	10	11	12	13	14	15	16	17	18	19	20	21	22	23	24	25	26	27	28	29	30	31	32	33	34	35	36
Protocol preparation: management of authorizations, submission to the research ethics committee, logistics, and development of data collection documentation																																				
**Phase 1.** Fieldwork: patient recruitment and data collection																																				
**Phase 1.** Data tabulation, variable coding, and data analysis																																				
**Phase 1.** Transcription of qualitative data and content analysis																																				
**Phase 1.** Interpretation, writing, and dissemination of Phase 1 results: manuscript submission to journals and academic defense																																				
**Phase 2.** Bibliographic review and concordance of the evidence with Phase 1 results																																				
**Phase 2.** Operationalization of interventions																																				
**Phase 2.** Development of the procedure																																				
**Phase 3.** Participant Recruitment and Consensus Conference: Drafting of the Official Record																																				
**Phase 3.** Development of the Final Procedure																																				
**Phase 3.** Dissemination of results																																				
